# Aloe Vera/Collagen Mixture Induces Integrin α1β1 and PECAM-1 Genes Expression in Human Adipose-Derived Stem Cells

**DOI:** 10.15171/apb.2019.077

**Published:** 2019-10-24

**Authors:** Faraz Sigaroodi, Hajar Shafaei, Mohammad Karimipour, Mohammad Amin Dolatkhah, Abbas Delazar

**Affiliations:** ^1^Stem Cells Research Center, Tabriz University of Medical Sciences, Tabriz, Iran.; ^2^Department of Anatomical Sciences, Faculty of Medicine, Tabriz University of Medical Sciences, Tabriz, Iran.; ^3^Faculty of Pharmacy and Drug Applied Research Center, Tabriz University of Medical Sciences, Tabriz, Iran.

**Keywords:** Adipose-derived stem cells, Aloe vera gel, Collagen, Integrin, PECAM-1, Tissue engineering

## Abstract

***Purpose:*** Natural biomaterials are a key base in tissue engineering, and collagen, as the main content of the extracellular matrix (ECM), is frequently used in tissue engineering. Aloe vera has some therapeutic effects on ulcers, therefore, the use of this natural resource has always been considered for improving collagen function. We aimed to evaluate the effect of Aloe vera/ Collagen blended on cell viability, cell attachment, and angiogenic potential by determining of integrin α1β1 and platelet endothelial cell adhesion molecule (PECAM-1) genes expression in human adipose-derived stem cells (hASCs).

***Methods:*** In this study, hASCs after harvesting of adipose tissues from abdominal subcutaneous adipose tissue and isolation, were cultured in four groups of control, collagen gel, Aloe vera gel, and Aloe vera/collagen blended in vitro environment at 24h and then cell viability was assessed by MTT (3-(4,5-dimethylthiazol 2-yl)-2,5-diphenyltetrazolium) assay. Integrin α1β1 and PECAM-1 genes expression were evaluated by real-time RT-PCR.

*** Results: ***The results of MTT showed that the combination of Aloe vera/collagen was retained the cell viability at the normal range and improved it. In real-time RT-PCR results, integrin α1β1 and PECAM-1 gene expression were increased in the Aloe vera/collagen blended group compared to the control group.

***Conclusion:*** For tissue engineering purposes, Aloe vera improves collagen properties in the culture of hASCs by increasing the expression of the integrin α1β1 and PECAM-1 genes.

## Introduction

In tissue engineering, natural materials are attractive for researchers due to cell adhesion, proliferation, and migration properties during the tissue construction or repair process. When these materials are located in a defective area, derived biological substances can naturally increase the migration of cells and integration of them to the surrounding environment, thus forming an extracellular matrix (ECM) and improving the structure of the repaired tissue.^1^

Collagen as a natural biomaterial is one of the main and most important ECM proteins and is considered as an ideal scaffold or matrix in tissue engineering.^2^ In addition, in the collagen fibril formation, cell surface receptors including integrins and cell adhesion compounds contribute to collagen polymerization.^3^ Integrins play important roles in wound healing and help to genes expression associated with this process.^4^ Talin as an intracellular protein, upregulates integrin α1β1 in endothelial cells, smooth muscle cells, fibroblasts, and other cell types.^5^ Another important cell surface marker, platelet endothelial cell adhesion molecule (PECAM-1or CD31), has essential role in the cell invasion and migration through the collagen matrix. PECAM-1 seems to be urgent for cell elongation, migration, and invasion in the gels; as well as it is noticeable for cell-cell assembly to form the endothelial cells network structures.^6^ However, some disadvantages of collagen material such as insufficiency in the control of infection, contraction of transplanted tissue in defect area and changes in cell growth^7,8^ have resulted that researchers attempt to find appropriate materials for combination with collagen to solve mentioned problems.

Aloe vera (a member of the Liliaceous family) is a tropical plant that has a reparative potential for various types of soft tissue damage. Aloe vera gel include more than 98%-99% water and 60% of its dry matter are polysaccharides. It is believed the usefulness of Aloe vera in the treatment of disorders such as arthritis, gout, acne, dermatitis, and wounds such as stomach ulcers and burns.^9,10^ Angiogenesis is a necessary mechanism for wound healing and β-sitosterol, as the main compound of Aloe vera gel, was induced strong angiogenic effects in the chick chorioallantoic membrane (CAM) assay and Matrigel plug assay by human endothelial cells^11^ and also It was found that Aloe vera increases the collagen type III content in repaired tissue as well.^12^

Adipose tissue has been demonstrated as an ideal source of adult tissue-derived stem cells with amazing properties for tissue engineering and regenerative medical procedure. Human adipose-derived stem cells (hASCs) differentiate in vitro to adipocytes, cardiac myocytes, smooth muscle, osteocytes, and chondrocytes when treated with specific factors.^13^ Considering the popularity of hASCs due to some advantages such as accessibility, and the importance of integrin α1β1 and PECAM-1 in viability, proliferation, migration and cellular differentiation in the context of tissue engineering, this study designed to evaluate the effects of freshly Aloe vera gel added to collagen gel on hASCs.

## Materials and Methods

### Collagen extraction

In this study, type I collagen was extracted from the tail tendons of the rat according to the previously published method.^14^ For sterilization of collagen, we carefully transferred the solution to a bottle with a screw cap and containing CHCl_3_ (Chloroform) - 10% of the volume of extracted collagen - at the bottom and allowed to rest 12 h at 4°C and then aseptically removed the collagen solution.^15^

### Aloeveragel preparation

Aloe vera gel was extracted from fully mature Aloe vera plant with a little change in the previously published method.^16^ In brief, the mature leaves were removed after surface washing, and their shell was removed in clean conditions. White pulp Aloe vera gel was homogenized by a mixer and centrifuged at 12 000 rpm for 30 min at 4°C to divide the fibers. Sterilization of Aloe vera gel was performed the same as collagen gel.

### Cell culture

Adipose tissues were acquired from abdominal subcutaneous adipose tissue.^17^ According to Zuk protocol^18^ Isolation of hASCs performed by collagenase A (Worthington-CSLAFA) Briefly, hASCs were cultured at 37°C, 5% CO2 in low glucose DMEM (Gibco, UK) and also 10% placental serum.^19^ After 24h, the culture media was replaced, then the culture medium is changed every 2–3 days. During the cell culture, the morphology and proliferative potentiality of hASCs were determined microscopically. The cells were trypsinized (0.25% trypsin/0.2% EDTA; Sigma, USA) and the 3th passages of harvested cell suspension was used in groups of study.

### Fabrication of Aloevera/Collagen blended

For preparation a blend of Aloe vera and collagen, under a laminar flow hood and about 4°C, by keeping a constant volume of blend, 30% Aloe vera gel was blended with 70% collagen gel by pipetting at the glasses dish. Collagen gel, Aloe vera gel, and their blend distributed on 6-well culture plates after adjusting pH in 7.4.^14^ Four groups of hASCs were cultured on collagen gel, Aloe vera gel, Aloe vera/collagen blended, and hASCs without any gel. The cells of all groups were grown to confluence.

### Cell viability evaluation

The cells of all groups were cultured in 96-well plates (3000 cells per well), after 24 h, culture media was removed and 200 µL serum-free DMEM medium and 20 µL of MTT solution (5 mg/mL, Sigma USA) were added onto the cells. After four hours, 100µL of dimethyl sulfoxide (DMSO, Sigma, Germany) was added onto the chambers to dissolve the formazan crystals. After 30 min in a dark room, the optical density was recorded by ELISA reader at 540 nm.

### RNA extraction, cDNA generation, and quantitative reverse transcriptase polymerase chain reaction (real-time RT-PCR)

The RNX-Plus solution (Phenol + Guanidine Isothiocyanate) for total RNA extraction was the product of SinaClon (Cat.no: PS4131). In brief, 24 h after treatment, the medium culture was removed from groups and the Total RNA of hASCs was extracted using the RNX- Plus Solution kit, according to the company manual. After purification and quantification, RNA was determined by measuring the optical density at 260 and 280 nm using Nanodrop (Nanodrop- ND-1000). PrimeScript reagent kit (Cat.no: RR037Q) for cDNA synthesis, was purchased from Takara Inc. Real Q Plus 2x and the first strand of cDNA was generated from 500 ng of extracted total RNA using the Takara PrimeScript reagent kit according to the protocol provided by the manufacturer. Master Mix Green High ROX was purchased from Ampliqon (Cat.no: A 235402). SYBER green and ROX were used as the reporter and reference dies respectively and the relative amount of mRNA for each target was normalized to the gene expression. Gene-specific primer sets used in this study are shown in Table 1. For statistical analysis, the results were presented as means ± SEM. Statistical differences between different groups were tested by One-way analysis of variance (ANOVA) using Graph Pad Prism software. *P* < 0.05 was determined as significant.

**Table 1:** Gene-specific primer sets used for real-time RT-PCR

ITGA1-F	5’-CGGTACAATCATACAGGCCA-3’
ITGA^1^-R	5’-TTGCTCCTCCTTCTCTGTTC-3’
ITGB1-F	5’-AATGCCTACTTCTGCACGAT-3’
ITGB1-R	5’-GCTTCTCTGCTGTTCCTTTG-3’
PECAM1-F	5’-CTGGGAGGTCGTCCATGT-3’
PECAM1-R	5’-CACAGGACTCTCGCAATCC-3’
GAPDH F	5’-CCTGCACCACCAACTGCTTA-3’
GAPDH R	5’-GGCCATCCACAGTCTTCTGAG-3’

## Results and Discussion

### Normal morphological appearance ofhASCsin presence Aloeveragel and collagen gel

**Figure 1:**
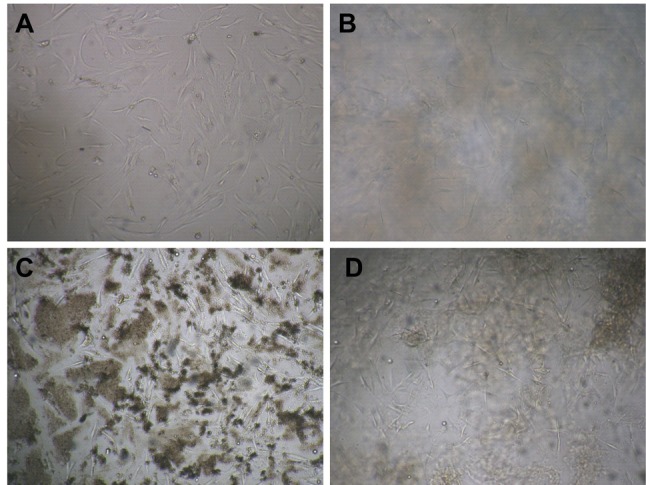
Morphology of ACSs at 24 h. A) Cell, B) Collagen gel, C) Aloe vera gel, D) A/C (magnification, ×40)

After 24 h of hASCs culture with Aloe vera and its combination with collagen, we observed that normal cell proliferation and cell morphology in treated hASCs versus control group (Figure 1). hASCs have fibroblast-like morphology and lack lipid droplets in the cytoplasm.^20^ Our microscopy observations demonstrated that hASCs with Aloe vera/collagen blended were morphologically similar to those grown in control group and also homogeneous morphology was observed in control and treated groups. Phenotypic durability and homogeneity is very important in clinical cell research and tissue engineering, and should be guaranteed.

### Cell viability effect of AloeveraonhASCs

**Figure 2:**
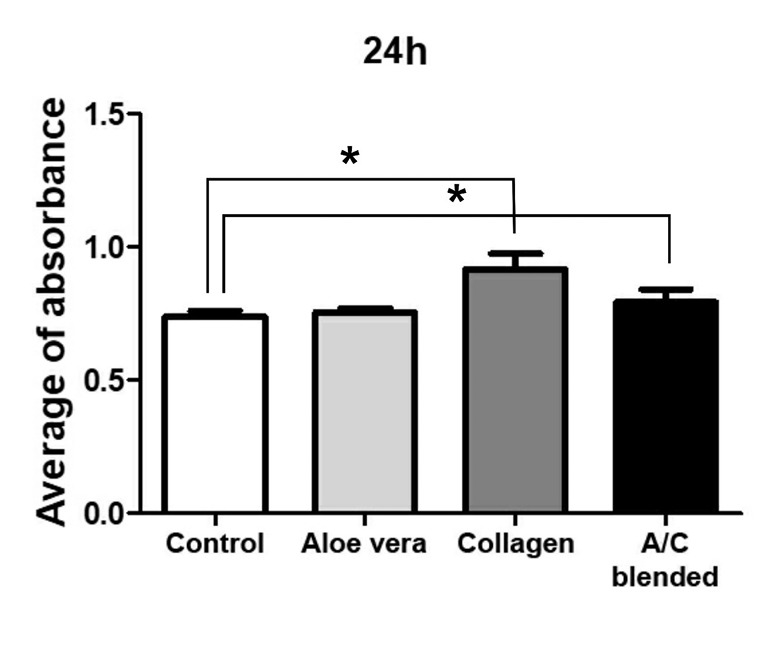
Influence of collagen gel, Aloe vera gel and Aloe vera/collagen blended (A/C) on the proliferation of ASCs after 24hours. Cell viability was determined by MTT assay. ASCs cell viability rate in control The graph in A/C has shown an increase compared to control and collagen significantly increased then A/C. Error bars represent the SEM (*P < 0.05).

Viability effect of materials is a very important factor in tissue engineering studies. The viability of treated cells was evaluated by MTT assay. The results were compared with the control group that included only hASCs as shown in Figure 2. In Aloe vera/Collagen blended group, the numbers of hASCs were significantly high compared to control. In recent years, many interesting results were achieved regarding the proliferative properties of stem cells. High proliferative capacity of stem cells can produce a large number of cells to regenerate tissue defects. The proliferation potentiality of adipose-derived stem cells (ASCs) seems to be higher than other mesenchymal stem cells such as bone marrow-derived. Previous reports have shown that the doubling times of ASCs during the logarithmic phase of growth range from 40 to 120 h.^21-23^ Cell growth was observed in the presence/absence of Aloe vera in the scaffolds and was founded that collagen-chitosan-Aloe vera is better than collagen-chitosan.^16^ In our study, results in all groups after 24 hours showed normal proliferation and increased in Aloe vera/collagen blended. Therefore, Aloe vera enhances the biological properties of collagen for hASCs and this blend naturally increases the cell viability rate of hASCs. These findings have been consistent with Lee et al reports.^24^ Their studies showed that Aloe vera gel strongly increased the proliferation of calf pulmonary artery endothelial cells.

### Aloevera/collagen blended (A/C) enhance the gene expression levels of ITGA1, ITGB1, and PECAM-1 inhASCs

**Figure 3:**
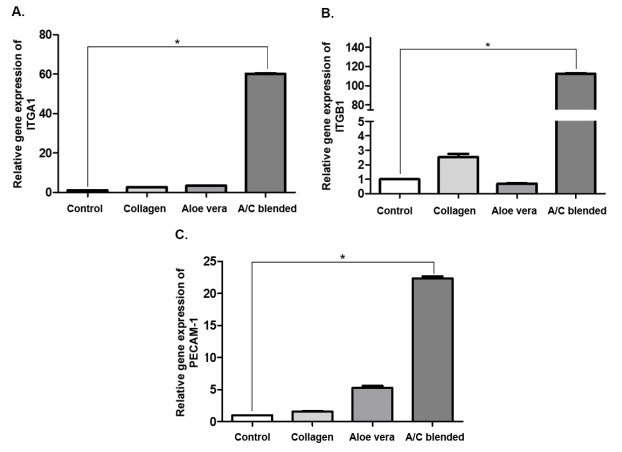
Effects of Collagen gel, Aloe vera gel, and their blend on relative gene expression of ASCs by real time RT-PCR. A) ITGA1, B) ITGB1, C) PECAM-1, Error bars represent SEM (*P < 0.05).

It has been shown that integrins play an important role in the development, ECM structure, angiogenesis, and wound healing.^25-27^ we performed real-time RT-PCR analysis in order to evaluate the gene expression of two integrin subunits include ITGA1 and ITGB1. Our gene expression results analysis revealed that gene expression levels of ITGA1 and ITGB1 significantly (*P* <0.05) were enhanced in hASCs with Aloe vera/collagen blended (Figure 3A and 3B respectively). Also, in collagen gel group, ITGB1 notably was increased in comparison to control. Integrin α1β1 is usually presented in the mesenchymal cells and it is most abundant in vascular and visceral smooth muscle. Also, integrin α1β1 is one of the main factors in structure organization of the ECM with collagen regulation.^28-30^ Katz et al^31^ In their study on hASCs, shown these cells were able to express the α1 (ITGA1) and β1 (ITGB1) subunits genes. In addition, the use of Aloe vera has caused positive changes in the cell cycle, the process of gene expression of differentiation markers in human primary epidermal keratinocytes (HPEKs); HPEKs treated with Aloe vera gel or Cape aloe extract, significantly expressed higher levels of β1-integrin, β4-integrin, α6-integrin, and E-cadherin.^32^ In this study, we found that the Aloe vera/collagen blended was effective on the gene expression level of α1 and β1 subunits and it raises the amount of them compared to the control group.

**Figure 4:**
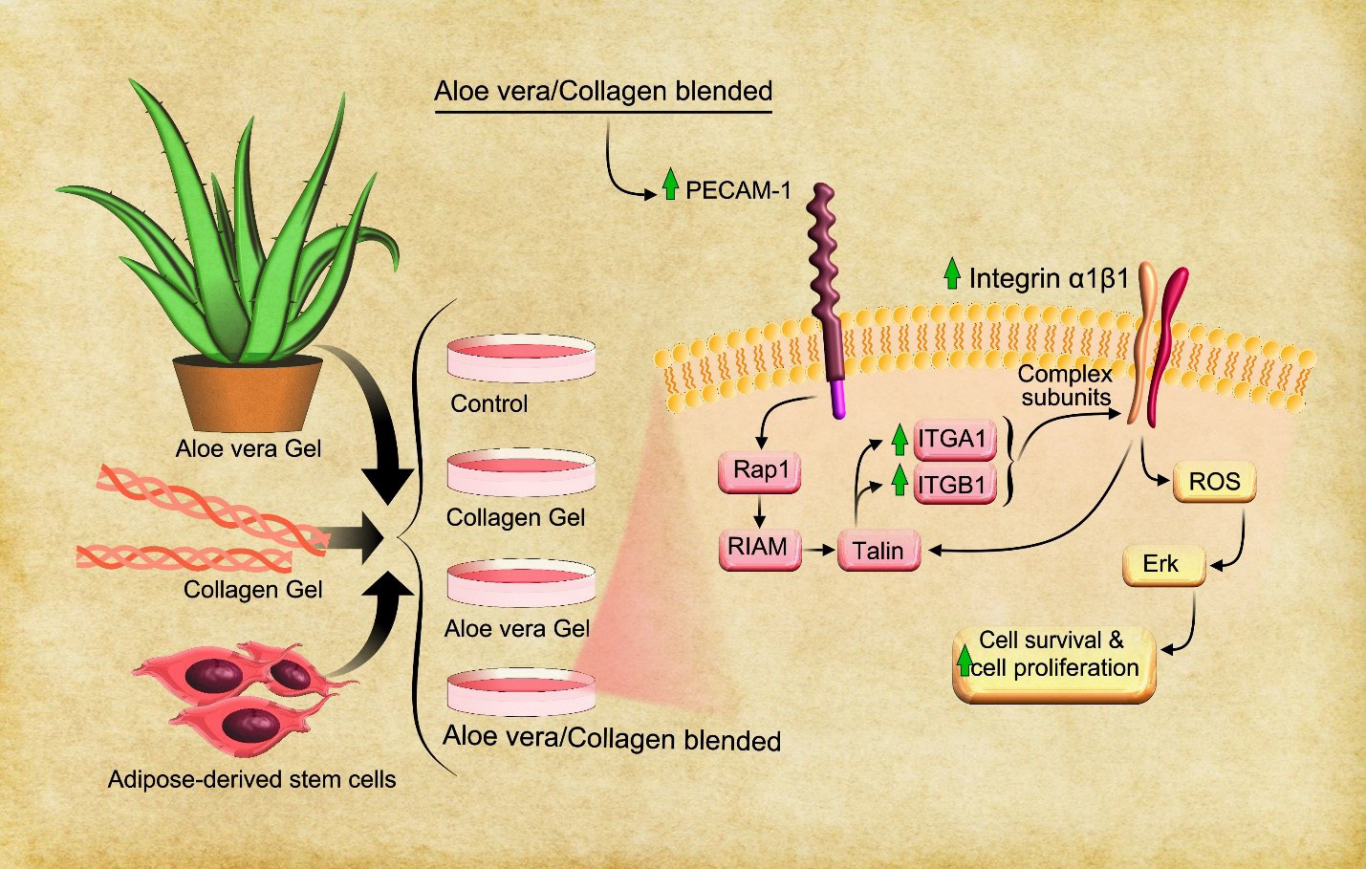
The effect of Aloe vera/Collagen blended on the PECAM-1 gene expression of ASCs and the possible pathway of integrin α1β1 induction by PECAM-1. Aloe vera and collagen gels, as well as adipose-derived stem cells, were extracted and studied in four groups; control, collagen gel, Aloe vera gel, and Aloe vera/Collagen blend. RT-PCR studies showed that the Aloe vera/Collagen blended group significantly increased the gene expression of integrin α1β1, and PECAM-1. This increase can be checked through the pathway induced by Talin and its role in promotion of integrin α1β1. Also, integrin in through the ROS pathway effects on cell survival and cell proliferation.

In agreement with previous studies, endothelial markers were expressed on ASCs such as PECAM1 (CD31) but hASCs showed low levels (<0.4%) gene expression of PECAM-1 at compared to primary endothelial cells.^33^ In fact, this gene expression is observed in the process of ASCs differentiation to vascular cells.^34^ Based on our results, hASCs in collagen group hasn’t shown augmentation in PECAM-1 gene expression. However, gene expression of PECAM-1 significantly (*P* < 0.05) increased in hASCs with A/C (Figure 3C), and due to the properties of Aloe vera and its essential component: β-sitosterol, in induction of angiogenesis as the main process of vascular development that has been evaluated in the past,^11^ can be concluded that the increase of PECAM-1 in our study with the capability of Aloe vera in differentiation stem cells is associated and may cause the Aloe vera/collagen blended to incite hASCs to angiogenesis. In addition, another study showed that PECAM-1 promotes many integrin subunits^35^ and this regulation is done through the path of Talin activation, that Ras-related protein 1 (Rap1) and Rap1–GTP-interacting adapter molecule plays a key role in this gene expression cascade, respectively.^36^ On the other hand, Talin regulates integrin α1β1.^37^ However, integrins are effective on cell viability by activating of reactive oxygen species (Figure 4).^35^ Finally, many studies have shown that integrin α1β1 heterodimers are involved in the regulation of cell growth, survival, migration, especially in the angiogenesis process^38^ and then increasing of PECAM-1. Our study introduces novel combined biomaterial by Aloe vera and collagen for tissue engineering due to Aloe vera effects on stimulating of cell adhesion, cell viability and hASCs differentiation toward angiogenesis process.

## Conclusion

In Conclusion, our study found that Aloe vera/Collagen blended can improves cell proliferation, cell attachment due to high expression of integrin α1β1, and stimulatory effect on PECAM-1. This study was performed in the short-term and it is needed to study by more evaluations in a long time.

## Ethical Issues

This study was performed under rules and principle of the ethical committee of Tabriz University of Medical. The ethical code of this research and using of tail tendons is 58535 and 58536, respectively.

## Conflict of Interest

There is no conflict of interests

## Acknowledgments

Authors indebted to the Stem Cells Research Center (Umbilical Cord Unit) of Tabriz University of Medical Sciences for Financial and scientific support of our study.
